# Figure of merit for ionic thermoelectric materials

**DOI:** 10.1093/nsr/nwag227

**Published:** 2026-04-20

**Authors:** Kang Zhu, Mao Yu, Yuchen Li, Shangchao Lin, Zhuoxin Liu, Dan Zhao, Nicholas X Fang, Xiaogang Zhang, Weishu Liu

**Affiliations:** School of Advanced Energy, Sun Yat-sen University, China; Department of Materials Science and Engineering, Southern University of Science and Technology, China; Department of Materials Science and Engineering, Southern University of Science and Technology, China; Department of Materials Science and Engineering, Southern University of Science and Technology, China; Department of Mechanical Engineering, The University of Hong Kong, China; Institute of Engineering Thermophysics, School of Mechanical Engineering, Shanghai Jiao Tong University, China; Guangdong Provincial Key Laboratory of New Energy Materials Service Safety, College of Materials Science and Engineering, Shenzhen University, China; Laboratory of Organic Electronics, Department of Science and Technology, Linköping University, Sweden; Department of Mechanical Engineering, The University of Hong Kong, China; Jiangsu Key Laboratory of Electrochemical Energy Storage Technologies, College of Material Science and Technology, Nanjing University of Aeronautics and Astronautics, China; Department of Materials Science and Engineering, Southern University of Science and Technology, China

## Abstract

A new performance index is proposed for ionic thermoelectric materials, to bridge the material properties and energy conversion efficiency in real-world application scenarios.

Ionic thermoelectric (i-TE) materials, based on thermodiffusion (TD) and/or thermogalvanic (TG) effects, hold the promise of energy harvesting with a much simpler structure and lower cost than electronic thermoelectric counterparts. Nevertheless, since ions cannot cross the electrodes into the external circuit, TD or TD–TG coupled i-TE power generators can only operate in a discontinuous mode, with separate stages including thermal charge, power output, and rest for ionic restoration, rather than a continuous mode for the electronic TE devices [1–3]. The past years have witnessed many efforts in the materials design, including the ion–matrix interaction [4–7], ion–ion interaction [8–10], and the confinement effect of ions [11,12] for TD cells, selective ion crystallization [13,14], and solvation structure regulation for TG cells [15]. They have further brought about tangible progress in increasing the energy output of the i-TE devices by enhancing the capacitance of TD cells [1,16], reducing the charge transfer resistance of TG cells [3,17] and the internal thermal resistance [13], enlarging the working temperature range by tuning the freezing [18], and melting temperatures [19] of the i-TE gels. However, a comprehensive physical picture and performance index that integrates all effects is still missing. In other words, there is still a lack of a figure-of-merit for the i-TE materials defined from the rigorous relation between the efficiency and material property parameters based on the thermodynamic consideration of the discontinuous working mode. We can’t directly borrow the figure of merit $ZT = \frac{{{S}^2\sigma T}}{\kappa }$ (*S*, $\sigma $, $\kappa $, and *T* denote the thermopower, electric and thermal conductivity, and absolute temperature, respectively), used in the electronic thermoelectric materials due to the different working mode. Ions accumulate on the surface of the cold electrode under the temperature gradient, providing a direct driving force to attract electrons across the external circuit for operation (i-TE generator mode), or as an essential component in forming an electrical double-layer capacitor for charge storage (i-TE capacitor mode). In other words, the DC-conductivity $\sigma $ is not yet a good scale for qualifying the ionic materials. Song *et al.* [20] have proposed a figure-of-merit for the i-TE capacitor mode by considering the work done by charge storage, that is, $ZT = \frac{{S_{td}^2}}{\kappa }\frac{{C{G}_T}}{{{\rho }_d{c}_p}}T$, where *S_td_, κ, C, ρ_d_, c_p_*, and *G_T_* are the thermopower, thermal conductivity, capacitance, density, specific heat, and interfacial thermal conductance, respectively. Although the second term has the unit of electrical conductivity, as compared with the e-TE figure-of-merit, its physical insight remains unclear. Furthermore, in their derivation, the diffusion characteristics of ions during the thermal-voltage buildup process were not explicitly accounted for. Qian *et al.* [21] proposed new figure of merits for both thermodiffusive devices as $Z = \frac{{CS_{td}^2}}{{K( {{t}_{ch} + 3{R}_LC} )}}$ and TG ones as $Z = \frac{{S_{tg}^2}}{{{R}_sK}}$, respectively, in which *C, S_td_, K, t_ch_*, and *R_L_* are the capacitance, thermodiffusive thermopower, device thermal conductance, thermal charging time, and the load resistance, and *S_tg_* and *R_S_* denote the TG thermopower and overall resistance of the device, respectively. The importance of reducing the thermal charging time to improve efficiencies for TD devices is highlighted, while TG devices are illustrated to behave similarly to electronic thermoelectric ones in the limit of $Bi \to \infty$, with $Bi$ denoting the Biot number, which quantifies the thermal coupling between the device and heat reservoir. In this short communication, we present new derivations of a figure-of-merit for i-TE materials (both TD and TD–TG coupled) based on the i-TE generator mode, fully accounting for the time-dependent voltage induced by ionic diffusion, and discuss a practical strategy to achieve maximum efficiency.

Under a stable temperature gradient, unlike electronic thermoelectric generators (e-TEG), which feature negligibly short charging times and nearly non-decaying voltage outputs, ionic thermoelectric generators (i-TEG), dominated by the TD effect and/or TG effect, typically exhibit much longer thermal charging times. Moreover, their voltage continuously decays during discharge, indicating capacitive behavior. The charge–discharge voltage variations of an e-TEG and an i-TEG over one operation cycle are illustrated in Fig. 1(a) and (b), respectively. First, we will start from a simplified model that regards the i-TE generator as a capacitor ${C}^*$, which accumulates electric potential and energy during the thermal charging stage and releases them during the electrical discharging stage through a load resistance ${R}_L$.

**Figure 1.**
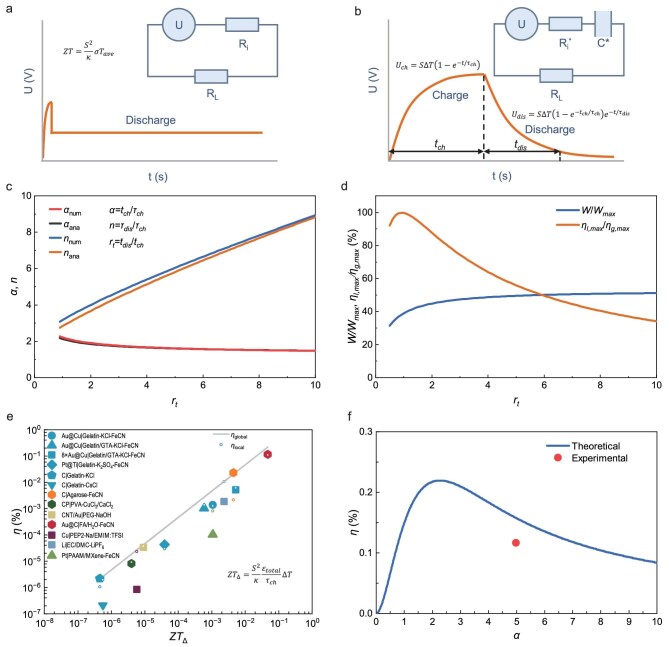
Modeling and evaluation of i-TE power generators. (a) An equivalent electric circuit of an electronic thermoelectric device and the voltage curve during the thermal charging and discharging stage. (b) An equivalent electric circuit of an i-TE device and the voltage curve during the thermal charging and discharging stages. (c) Comparison of the analytical to numerical results of ${n}^*$ and ${\alpha }^*$ in local optimization with ${r}_t$ ranging from 0.9 to 10. (d) Relative energy output and relative efficiency of local optimization vs. global optimization with ${r}_t$ ranging from 0.5 to 10. (e) Verification of the linear relationship between the proposed figure of merit $Z{T}_{\mathrm{\Delta }}$ and the energy conversion efficiency by previous experimental results of multiple i-TE material systems and electrode designs. (f) Comparison of the experimentally measured energy conversion efficiency of the FA/H_2_O-FeCN i-TE cell with the theoretical curve under the given thermal charging time.

The thermal charging process can be approximated by a standard capacitor charging behavior as


(1) \begin{eqnarray*}
{U}_{ch} = S{\mathrm{\Delta }}T\left( {1 - {e}^{ - t/{\tau }_{ch}}} \right),
\end{eqnarray*}

(2) \begin{eqnarray*}
{I}_{ch} = {C}^*\frac{{{\mathrm{d}}{U}_{ch}}}{{{\mathrm{d}}t}} = \frac{{{C}^*}}{{{\tau }_{ch}}}S\Delta T{e}^{ - t/{\tau }_{ch}},
\end{eqnarray*}

where *S* and ${\mathrm{\Delta }}T$ are the thermopower and temperature difference across the TD device, respectively, and the characteristic time is denoted as ${\tau }_{ch} = {R}_i{C}^*$, in which ${R}_i$ is the internal resistance of the cell. The cell voltage and current during the discharging process is then given by


(3) \begin{eqnarray*}
{U}_{dis} = S{\mathrm{\Delta }}T\left( {1 - {e}^{ - {t}_{ch}/{\tau }_{ch}}} \right){e}^{ - t/{\tau }_{dis}},
\end{eqnarray*}

(4) \begin{eqnarray*}
{I}_{dis} = \frac{{{C}^*}}{{{\tau }_{dis}}}S{\mathrm{\Delta }}T\left( {1 - {e}^{ - {t}_{ch}/{\tau }_{ch}}} \right){e}^{ - t/{\tau }_{dis}},
\end{eqnarray*}

where ${t}_{ch}$ is the thermal charging time and ${\tau }_{dis} = ( {{R}_i + {R}_L} ){C}^*$ characteristic time of the discharging process, with ${R}_L$ denoting the load resistance. After a detailed analysis of the heat flow across and power output of the device (Supplementary Data), we obtain the energy output of the cell during a single charging–discharging cycle as


(5) \begin{eqnarray*}
&& W = \frac{{n - 1}}{{2n}}{C}^*{\left( {{\mathrm{S}}\Delta T} \right)}^2{\left( {1 - {e}^{ - \alpha }} \right)}^2 \\ && \qquad \times \, \left( {1 - {e}^{ - \frac{{2{r}_t\alpha }}{n}}} \right),
\end{eqnarray*}

where ${r}_t = {t}_{dis}/{t}_{ch}$ is the ratio of discharging time to thermal charging time, $n = {\tau }_{dis}/{\tau }_{ch}$ and $\alpha = {t}_{ch}/{\tau }_{ch}$. Note that these non-dimensional parameters can be freely controlled in practical operations. According to Eq. (5), the maximum energy output of ${W}_{max} = 1/2{C}^*{( {{{S}}\Delta T} )}^2$ is achieved under sufficiently large *n*, $\alpha $, and ${r}_t\alpha /n$, in line with the typical behavior of a capacitor. However, spending excessive time on a single cycle to maximize the energy output is obviously unreasonable, as it will result in extremely low efficiency.

The heat to power conversion efficiency is given by


(6) \begin{eqnarray*}
\eta = {\eta }_C \cdot Z{T}_{\mathrm{\Delta }} \cdot F\left( {{r}_t,n,\alpha } \right),
\end{eqnarray*}

where ${\eta }_C = \frac{{{\mathrm{\Delta }}T}}{{{T}_h}}$ being the Carnot efficiency, $Z{T}_{\mathrm{\Delta }}$ being identified as a new figure of merit for i-TE materials as


(7) \begin{eqnarray*}
Z{T}_{\mathrm{\Delta }} = \frac{{{S}^2}}{\kappa }\frac{{{\varepsilon }_{\textit{total}}}}{{{\tau }_{ch}}}\Delta T,
\end{eqnarray*}

where $\kappa $ is the thermoelectric conductivity of the material, and ${\varepsilon }_{\textit{total}}$ is the equivalent permittivity. Note that the term ${\varepsilon }_{\textit{total}}/{\tau }_{ch}$ has the same unit of the electric conductivity, which might be named as a general conductivity. The general conductivity of ${\varepsilon }_{\textit{total}}/{\tau }_{ch}$ is a direct result of considering the power output as a supercapacitor. The ionic TD could contribute to non-Faradaic capacitance (that is, the charge displacement), while ionic TG effect plays as a Faradaic capacitance. Furthermore, TG cell with redox couples actually could work continuously in the liquid due to the mass convection, which consequently makes the general conductivity actually the same as a DC-conductivity. As a result, in an i-TEG device with both thermodiffusive ions and TG redox-couples, the term of ${\varepsilon }_{\textit{total}}/{\tau }_{ch}$ could be further expressed by the combination of thermodiffusive part $\varepsilon /{\tau }_{ch}$ and the TG part $\sigma $. This insight could be used to phenomenally guide the optimization of i-TEGs. It is worthy to point out that the general conductivity could be extracted from the voltage–time curve of the thermal charge and electrical discharge, and be used to calculate the $Z{T}_{\mathrm{\Delta }}$ and hence estimate the efficiency. However, the accurate separation of the thermodiffusive part $\varepsilon /{\tau }_{ch}$ and the TG part $\sigma $ from the total general conductivity is still very challenging, which needs more careful experimentation.

The third term at the right-hand side of Eq. (6) is a working decision function with an expression as follows:


(8) \begin{eqnarray*}
&& F\!\left( {{r}_t,n,\alpha } \right) \\ \!\!&& = \frac{{\frac{{n - 1}}{{2n}}{{\left( {1 - {e}^{ - \alpha }} \right)}}^2\left( {1 - {e}^{ - \frac{{2{r}_t\alpha }}{n}}} \right)}}{{\left( {1 + {r}_t} \right)\alpha {\eta }_C + Z{T}_{\mathrm{\Delta }}\left( {1 - {e}^{ - \alpha }} \right)\left( {2 - {e}^{ - \frac{{{r}_t\alpha }}{n}}} \right)}}. \\
\end{eqnarray*}

According to Eq. (8), once the available temperature difference, that is, the Carnot efficiency ${\eta }_C$, and $Z{T}_{\mathrm{\Delta }}$ are given, the energy conversion efficiency could find its maxima by tailoring ${r}_t$, *n*, and $\alpha $, enabling us to establish an operating guideline for efficient energy harvesting using i-TEs. By neglecting the very weak Peltier effect (the second term in the denominator of Eq. (8)) within the cell and letting $\partial F/\partial {r}_t = 0$, $\partial F/\partial n = 0$, and $\partial F/\partial \alpha = 0$, the optimal parameter combination is given by


(9) \begin{eqnarray*}
\left\{ {\begin{array}{@{}*{1}{c}@{}} {{e}^{\frac{{2{r}_t\alpha }}{n}} - 1 = 2\frac{1}{n}\left( {{r}_t + 1} \right)\alpha }\\ {{e}^{\frac{{2{r}_t\alpha }}{n}} - 1 = 2\left( {1 - \frac{1}{n}} \right){r}_t\alpha }\\ {\frac{{2\alpha }}{{{e}^\alpha - 1}} + \frac{{\frac{{2{r}_t\alpha }}{n}}}{{{e}^{\frac{{2{r}_t\alpha }}{n}} - 1}} = 1} \end{array}} \right..
\end{eqnarray*}

Numerically solving Eq. (9) reveals $r_t^* \approx 0.91$, ${n}^* \approx 3.1$, and ${\alpha }^* \approx 2.3$, and the globally maximum conversion efficiency is obtained as


(10) \begin{eqnarray*}
&& {\eta }_{g, max} = \frac{{0.203{\eta }_CZ{T}_{\mathrm{\Delta }}}}{{4.393{\eta }_C + 1.341Z{T}_{\mathrm{\Delta }}}} \\ &&\qquad\qquad \approx 0.046Z{T}_{\mathrm{\Delta }}.
\end{eqnarray*}

The two terms in the denominator in Eq. (10) account for the contributions to the total heat flux by thermal conduction and the Peltier effect, respectively. The ratio of the Peltier term to the conduction term is then $1.341Z{T}_{\mathrm{\Delta }}/( {4.393{\eta }_C} ) \approx 0.3Z{T}_h$. With a currently typical *Z* value of 10^–4^–10^–3^ as indicated by Table S1 in Supplementary Data, and a heat source temperature not too far from 300 K, the Peltier term is assessed to contribute to the total heat flux by 0.8%–8%, supporting the approximation made in the above equation. Equations (7) and (10) clearly indicate multiple ways to improve the performance of i-TE materials and devices including but not limited to promoting the thermopower. By using gel matrix with lower thermal conductivity (lowering $\kappa $), strengthening the matrix to withstand broader temperature range (enlarging ${\mathrm{\Delta }}T$), ordering the matrix framework to facilitate ionic transport (enlarging $\sigma $ while reducing ${\tau }_{ch}$) and tailoring micro/nanostructured interface of the electrodes (enlarging $\varepsilon $), the $Z{T}_{\mathrm{\Delta }}$ as well as the efficiency could be substantially improved. However, it should be always kept in mind that just like the case in electronic thermoelectrics, multiple interactions exist among the above parameters, and any active tuning of one parameter could be accompanied by changes in others.

It is now beneficial to return to the energy output per charging–discharging cycle. By substituting $r_t^*$, ${n}^*$, and ${\alpha }^*$ into Eq. (5), the energy output is $\sim 0.2{C}^{*}{( {{{S}}\Delta } T)}^2$, which is 40% of the maximum value that a single cycle could offer. This is possibly due to the relatively low $r_t^*$, that is, a short discharging time. In certain potential applications, it is often essential to trade off conversion efficiency and energy output in a single cycle. Here, we move a step further to find the locally maximum conversion efficiency (${\eta }_{l,\ max}$) condition under given $r_t^*$, which could find a wider application than the globally maximum efficiency. Letting $\partial F/\partial n = 0$ and $\partial F/\partial \alpha = 0$, the optimal combination of *n* and $\alpha $ is given by


(11) \begin{eqnarray*}
\left\{ {\begin{array}{@{}*{1}{c}@{}} {{e}^{\frac{{2{r}_t\alpha }}{n}} + \frac{{2{r}_t\alpha }}{n} = 1 + 2{r}_t\alpha }\\ {\frac{{2\alpha }}{{{e}^\alpha - 1}} + \frac{{\frac{{2{r}_t\alpha }}{n}}}{{{e}^{\frac{{2{r}_t\alpha }}{n}} - 1}} = 1} \end{array}} \right..
\end{eqnarray*}

Based on the numerical solutions of Eq. (11), a set of approximate analytical solution is given as


(12) \begin{eqnarray*}
\left\{ {\begin{array}{@{}*{1}{c}@{}} {n \approx \frac{{2.3{r}_t}}{{{\mathrm{ln}}\left( {1 + 1.25{r}_t} \right)}}}\\ {\alpha \approx \frac{{{\mathrm{ln}}\left( {1 + 6{r}_t} \right)}}{{{\mathrm{ln}}\left( {1 + 1.5{r}_t} \right)}}} \end{array}}, \right.
\end{eqnarray*}

and a comparison of the analytical to numerical results is illustrated in Fig. 1(c), which shows good agreements. Substituting Eq. (12) back into Eqs (5) and (8), we obtain the relative energy output $W/{W}_{max}$ and relative efficiency ${\eta }_{l, max}/{\eta }_{g, max}$ as a function of ${r}_t$ and illustrate them in Fig. 1(d). As ${r}_t$ increases from 0.91, $W/{W}_{max}$ realizes a rapid rise first and gradually saturates to 50%, while ${\eta }_{l, max}/{\eta }_{g, max}$ continuously drops. When ${r}_t = 3$, the energy output in a single cycle increases by 18% compared to that when ${r}_t = 0.91$, and the locally maximum efficiency retains 75% of the globally maximum value, so that a good balance can be made between the energy output and efficiency with a ${r}_t$ up to 3. In case of an intermittent heat source or a special maintenance scenario where only a single thermal charging–discharging cycle can be implemented during a relatively long period, the locally optimal operating mode should be adopted, considering its larger energy output in a single cycle. While under stably supplied heat source and automatically controlled operations, the globally optimal operating mode is preferred due to its higher efficiency.

Next, we present a verification of the linear relationship between the above figure of merits and the energy conversion efficiency, using available experimental results on the Gelatin-KCl-FeCN system [3,19,22], FA/H_2_O-FeCN system [18], PVA-CuCl_2_/CaCl_2_ system [23], Gelatin-CsCl system [24], PEP2-Na/EMIM: TFSI system [25], EC/DMC-LiPF_6_ system [26], Gelatin-K_2_SO_4_-FeCN system (unpublished), Agarose-FeCN system (unpublished), Gelatin-KCl system (unpublished), PEG400-NaOH system (unpublished), and PAAM/MXene-FeCN system (unpublished), covering both hydrogel matrix and aqueous solution based devices with different TD–TG couples (including single TD and TG systems) and various electrode designs. First, we need to evaluate involved parameters (*S*, $\varepsilon $, ${\tau }_{ch}$, and $\sigma $) in the $Z{T}_{\mathrm{\Delta }}$ formula based on the recording data during tests, and an established evaluation procedure is provided in Supplementary Data. The evolution of energy conversion efficiency vs. $Z{T}_{\mathrm{\Delta }}$ is shown in Fig. 1(e), which well follows the globally maximum efficiency curve according to Eq. (10), and agrees better with the locally maximum efficiency evaluations following Eqs (6), (8), and (12), convincingly supporting the proposed figure of merit for i-TE materials and maximum efficiency formula in this work. For the C|Agarose-FeCN i-TE system denoted in orange color, the measured efficiency is found to agree well with the globally maximum value while surpass the locally maximum one given by the present model, which could be caused by the current deficiency of the model in describing purely TG-based i-TE generators due to their potentially continuous working mode, and this deficiency will be carefully considered in our future work. As more specifically shown in Fig. 1(f), under the given thermal charging time of 1200 s ($\alpha = {t}_{ch}/{\tau }_{ch} \approx 5$), the experimental value of the thermoelectric conversion efficiency of FA/H_2_O-FeCN is lower than the theoretical curve, which is drawn by keeping *n* and ${r}_t$ at their globally optimal values while varying $\alpha $. This is reasonable since in our previous studies, a unified discharging time of 2 h (with ${r}_t \ge 3$) was adopted to realize high energy output rather than maximizing energy conversion efficiencies. The most important advantage of the newly proposed figure of merit $Z{T}_\Delta $ when comparing to existing ones given by Song *et al.* [20] and Qian *et al.* [21] lies in its compatibility with different types of i-TE systems. It is not only able to evaluate single i-TE effect based i-TE materials and cells, but also able to evaluate the more promising TD–TG coupled ones with synergistic performances, leading to a broader range of applications. Finally, it is worth pointing out that the concerns relative to the corrosion reaction between the non-inert metal electrodes and ionic solution or gels also deserve careful attention. In our previous works, the Au-coated Cu electrode were found to negligibly affect the thermovoltage and power output of Gelatin-KCl-FeCN based i-TE cells during the first few thermal charging-discharging cycles [3,19], as indicated by the reasonable agreement between analytically predicted efficiencies and experimental data in Fig. 1(e). While the corrosion of electrodes did cause a slow rise of the internal resistance and thus a reduction in power output after long-time operating cycles. For a long-term stable operation, inert electrodes in absence of corrosions are highly required for ionic thermoelectric devices.

In conclusion, we present a theoretical analysis of the voltage and current variations during the thermal charging and discharging processes of both TD-driven and TD-TG coupled i-TE modules, and identify $Z{T}_{\mathrm{\Delta }} = \frac{{{S}^2}}{\kappa }\frac{{{\varepsilon }_{\textit{total}}}}{{{\tau }_{ch}}}{\mathrm{\Delta }}T$ as a figure of merit which is linearly correlated with the energy conversion efficiency, with ${\varepsilon }_{\textit{total}}/{\tau }_{ch}$ being a generalized conductivity contributed by both the ionic TD and the TG effect. We further confirm the linear relationship between $Z{T}_{\mathrm{\Delta }}$ and the conversion efficiency among several types of i-TE cells with different electrode designs in our previous works. Moreover, the proposed analytical framework offers an executable operating strategy to achieve a good balance between the energy output and conversion efficiency, which is helpful to guide the design of i-TE materials and devices with better performance.

## Supplementary Material

nwag227_Supplemental_File
