# Supplementary material for: Figure of merit for ionic thermoelectric materials
Source: Natl Sci Rev. 2026 Apr 20;13(11):nwag227. doi: 10.1093/nsr/nwag227 (PMC13248875; doi:10.1093/nsr/nwag227)
Supplement: nwag227_Supplemental_File [file nwag227_supplemental_file.pdf]

Supplementary data for

Figure of merit for ionic thermoelectric materials

Kang Zhu[1,2,†], Mao Yu[2,†], Yuchen Li[2,6,†], Shangchao Lin[3], Zhuoxin Liu[4], Dan Zhao[5], Nicholas X. Fang[6], Xiaogang Zhang[7] and Weishu Liu[2,*]

[1]School of Advanced Energy, Sun Yat-sen University, Shenzhen, Guangdong 518107, China; [2]Department of Materials Science and Engineering, Southern University of Science and Technology, Shenzhen, Guangdong 518055, China; [3] Institute of Engineering Thermophysics, School of Mechanical Engineering, Shanghai Jiao Tong University, Shanghai 200240, China; [4]Guangdong Provincial Key Laboratory of New Energy Materials Service Safety, College of Materials Science and Engineering, Shenzhen University, Shenzhen, Guangdong 518055, China; [5]Laboratory of Organic Electronics, Department of Science and Technology, Linköping University, Norrköping SE-601 74, Sweden; [6]Department of Mechanical Engineering, The University of Hong Kong, Hong Kong, China; [7]Jiangsu Key Laboratory of Electrochemical Energy Storage Technologies, College of Material Science and Technology, Nanjing University of Aeronautics and Astronautics, Nanjing 211106, China

[*]Corresponding author. E-mail: liuws@sustech.edu.cn

[†]Equally contributed to this work.

1. Power and heat flow analysis of a i-TEG device

By analogy with the electronic thermoelectric effect, the heat flow rate during the thermal charging stage is given by

$$q_{ch} = \kappa A \frac{\Delta T}{d} + S T_h I_{ch} \tag{S1}$$

where the first term represents the thermal conduction part, and the second represents the ionic-induced Peltier effect. The electric current is written as

$$I_{ch} = C^* \frac{\mathrm{d}u_{C,ch}}{\mathrm{d}t} = \frac{C^*}{\tau_{ch}} S \Delta T e^{-t/\tau_{ch}} \tag{S2}$$

where $C^* = \frac{\varepsilon_{total} A}{d}$ denotes the capacitance of the device, with A and d representing the cross-sectional area and thickness of the device, respectively. $\tau_{ch} = R_i C^*$ is the characteristic time of the thermal charging stage, in which R_i is the internal resistance of the module. Denoting the duration of the thermal charging stage by t_{ch}, the total heat flow across the generator during the thermal charging stage is then

$$Q_{ch} = \int_0^{\delta} q_{ch} \mathrm{d}t = \kappa A \frac{\Delta T}{d} t_{ch} + C^* S^2 T_h \Delta T \left(1 - e^{-t_{ch}/\tau_{ch}}\right) \tag{S3}$$

The discharging stage starts with an initial voltage of $U_{ch,t_{ch}} = S\Delta T\left(1 - e^{-t_{ch}/\tau_{ch}}\right)$ provided by the thermal charging stage, and the following decline is given by

$$U_{dis} = S\Delta T\left(1 - e^{-t_{ch}/\tau_{ch}}\right) e^{-t/\tau_{dis}} \tag{S4}$$

where $\tau_{dis} = (R_i + R_L)C^*$ is the characteristic time of the discharging process and R_L is the load resistance. Letting $R_L = mR_i$, then $\tau_{dis} = (1+m)\tau_{ch}$. The electric current flowing through the load is

$$I_{dis} = \frac{C^*}{\tau_{dis}} S\Delta T\left(1 - e^{-t_{ch}/\tau_{ch}}\right) e^{-t/\tau_{dis}} \tag{S5}$$

Accordingly, the instant power and heat flow rate during the discharging stage are written as

$$P = I_{dis}^2 R_L = \frac{m}{(1+m)^2} \frac{(S\Delta T)^2 \left(1 - e^{-t_{ch}/\tau_{ch}}\right)^2}{R_i} e^{-2t/\tau_{dis}} \tag{S6}$$

$$q_{dis} = \kappa A \frac{\Delta T}{d} + S T_h I_{dis} \quad \text{(S7)}$$

By integrating Eqs. S6 and S7, the electric energy output and heat flow during the discharging stage is obtained as

$$W = \frac{m}{2(1+m)} C^* (S\Delta T)^2 \left(1 - e^{-t_{ch}/\tau_{ch}}\right)^2 \left(1 - e^{-2t_{dis}/\tau_{dis}}\right) \quad \text{(S8)}$$

$$Q_{ch} = \kappa A \frac{\Delta T}{d} t_{dis} + C^* S^2 T_h \Delta T \left(1 - e^{-t_{ch}/\tau_{ch}}\right)\left(1 - e^{-t_{dis}/\tau_{dis}}\right) \quad \text{(S9)}$$

where t_{dis} is the discharging time. Dividing the electric work by the total heat flow during the whole thermal charging-electrical discharging process, the energy conversion efficiency is obtained as

$$\eta = \frac{\frac{m}{2(1+m)} C^* (S\Delta T)^2 \left(1 - e^{-t_{ch}/\tau_{ch}}\right)^2 \left(1 - e^{-2t_{dis}/\tau_{dis}}\right)}{\kappa A \frac{\Delta T}{d}(t_{ch} + t_{dis}) + C^* S^2 T_h \Delta T (1 - e^{-t_{ch}/\tau_{ch}})(2 - e^{-t_{dis}/\tau_{dis}})} \quad \text{(S10)}$$

From Eqs. (S8) and (S10) it can be noted that the tailoring of the charging and discharging time is of importance to achieve a high energy output and efficiency. To facilitate analysis, a set of nondimensional parameter is introduced as $r_t = t_{dis}/t_{ch}$, $n = \tau_{dis}/\tau_{ch}$ and $\alpha = t_{ch}/\tau_{ch}$. According to the previous derivation, $n = 1 + m$. Then Eqs. (S8) and (S10) are rewritten as

$$W = \frac{n-1}{2n} C^* (S\Delta T)^2 (1 - e^{-\alpha})^2 \left(1 - e^{-\frac{2r_t\alpha}{n}}\right) \quad \text{(S11)}$$

$$\eta = \frac{\frac{n-1}{2n} C^* (S\Delta T)^2 (1 - e^{-\alpha})^2 \left(1 - e^{-\frac{2r_t\alpha}{n}}\right)}{\kappa A \frac{\Delta T}{d} \alpha (1 + r_t)\tau_{ch} + C^* S^2 T_h \Delta T (1 - e^{-\alpha})\left(2 - e^{-\frac{r_t\alpha}{n}}\right)} \quad \text{(S12)}$$

Rearranging Eq. (S12), we arrive at

$$\eta = \eta_C \cdot ZT_\Delta \cdot F(r_t, n, \alpha) \quad \text{(S13)}$$

where $\eta_C = \frac{\Delta T}{T_h}$ is the Carnot efficiency, ZT_Δ is identified as a new figure of merit for ionic thermoelectric materials as

$$ZT_\Delta = \frac{S^2}{\kappa} \frac{\varepsilon_{total}}{\tau_{ch}} \Delta T \quad \text{(S14)}$$

where ε_{total} is identified as the equivalent permittivity of the generator and the term $\varepsilon_{total}/\tau_{ch}$ a generalized conductivity, and

$$F(r_t, n, \alpha) = \frac{\frac{n-1}{2n} (1 - e^{-\alpha})^2 \left(1 - e^{-\frac{2r_t\alpha}{n}}\right)}{(1 + r_t)\alpha\eta_C + ZT_\Delta (1 - e^{-\alpha})\left(2 - e^{-\frac{r_t\alpha}{n}}\right)} \quad \text{(S15)}$$

2. Evaluation of ZT_Δ by a set of thermal charging-discharging experiment

Here we present a procedure for evaluating the involved parameters (S, ε_{total}, and τ_{ch}) in the ZT_Δ formula by a set of thermal charging-discharging experiment, to give a quantitative evaluation of ZT_Δ. Assuming the thermal charging and discharging curves can be fitted by exponential functions with different characteristic times, then the thermopower S and characteristic time τ_{ch} of the charge process can be simultaneously determined by fitting the voltage curve of the i-TE generator during a thermal charging process, as illustrated in Fig. S1(a). With a fitting function of $u_{ch} = u_0 + u_1 e^{-t/\tau_{ch}}$, τ_{ch} is directly obtained and S is given by $S = u_{ch,end}/\Delta T$.

The equivalent permittivity ε_{total} is obtained from the discharging curves as shown in Fig. S1(b)

and (c). With an integration of the current over time, the quantity of electric charge released by the i-TE generator is obtained as $q = \int_0^{t_{dis}} I\mathrm{d}t$, and ε_{total} is given by

$$\varepsilon_{total} = qd/AU_{dis,0} \tag{S16}$$

where d, A and $U_{dis,0}$ are the thickness, cross-sectional area and initial discharging voltage of the i-TE generator, respectively.

To guarantee experimental consistency and reliability when extracting critical parameters, several actions should be carefully taken. First, the i-TE cell shall be packaged in a standardized procedure to prevent excessive fluctuations in dimensions and the existence of any void within the cell. Second, the i-TE cell shall be thoroughly discharged before a new test cycle begins, to avoid electric potential residual within the cell. Third, the temperature difference as well as the specific temperature range imposed on the i-TE cell shall be stably maintained, since the ionic transport within the cell as well as the reaction rates of the redox couples at the electrodes are strongly affected by temperature. Suitable thermal interfacial materials could be adopted at the interfaces between the cell and the heat source/sink to suppress temperature deviations.

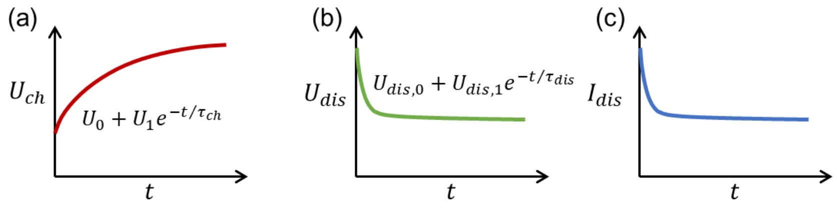

Fig. S1 Determination of the involved parameters from thermal charging-discharging experiments. (a) The thermal charging voltage curve. (b) The discharging voltage curve. (c) The discharging current curve.

3. Verification of the linear relationship between ZT_Δ and energy conversion efficiency

Thermal charging-discharging curves of various i-TE material systems are presented in Fig. S2, along with evaluated parameters given in Table S1, to verify the linear relationship between ZT_Δ and energy conversion efficiency.

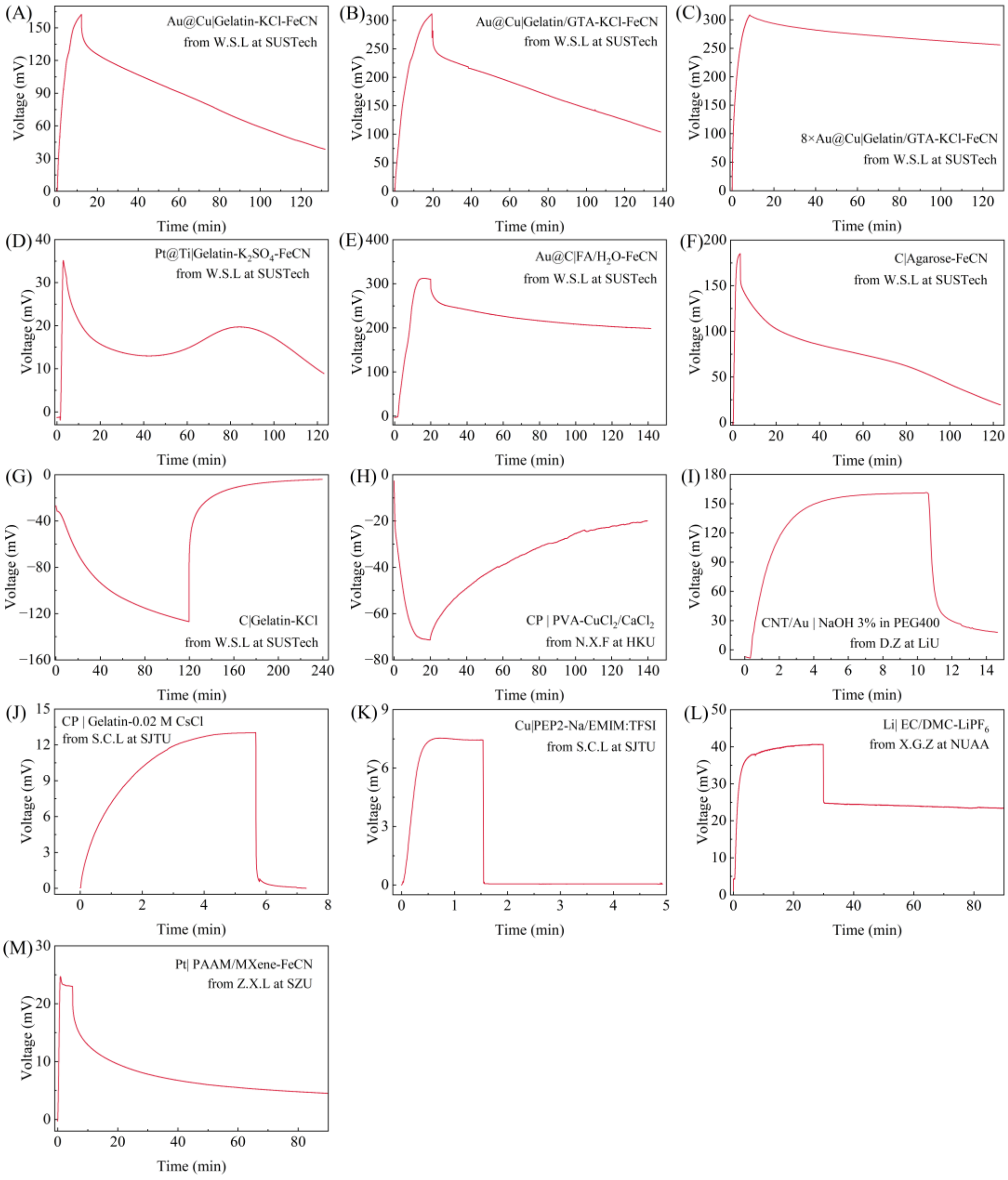

Fig. S2 Thermal charging-discharging curves of various i-TE material systems

Table S1 Test data and evaluated ZT_{Δ} of several i-TE material systems and electrode designs

**Electrode|i-TE material system**	S (mV K^{-1})	ε (F m^{-1})	τ_{ch} (s)	σ (S m^{-1})	κ (W m^{-1} K^{-1})	ΔT (K)	ZT_{Δ} (×10^{-3})	d (mm)	t_{ch} (s)	t_{dis} (s)	E_{2h} (J m^{-2})	Q_{tot} (MJ m^{-2})	η (%)
Au@Cu|Gelatin-KCl-FeCN [1]	17	10.4	166.5	0.013	0.15	9	**1.08**	1.8	500	7200	80	5.76	**1.39 ×10^{-3}**
Au@Cu|Gelatin/GTA-KCl-FeCN [2]	14.4	9.6	425.1	0.014	0.18	23	**0.6**	1.8	1200	7200	198	19.32	**1.02 ×10^{-3}**
8×Au@Cu|Gelatin /GTA-KCl-FeCN [3]	13.6	33.7	151.7	0.029	0.18	23	**5.25**	4	500	7200	403	7.95	**5.07 ×10^{-3}**
Pt@Ti|Gelatin-K_2SO_4-FeCN (unpublished)	10	6.6	839.7	0.005	0.14	7	**0.04**	2	300	7200	1.6	3.67	**4.35 ×10^{-5}**
Au@C|FA/H_2O-FeCN [4]	2.95	3257.89	240.64	7.336	0.26	105	**47.7**	10	1200	7200	27000	23.13	**0.12**

C|Agarose-FeCN (unpublished)	3.7	408.58	154.6	4.46	0.6	50	**4.52**	10	180	7200	3425	14.77	**0.023**
C|Gelatin-KCl (unpublished)	17	0.1464	2301.3	3.00×10^{-4}	0.28	7	**0.00046**	2	1200	7200	0.18	8.23	**2.19×10^{-6}**
CP | PVA-$CuCl_2$/$CaCl_2$ [5]	7.2	1.565	297.3	0.076	0.66	10	**0.0041**	3	1164	7200	1.48	18.4	**8.05×10^{-6}**
CNT/Au | NaOH 3% in PEG400 (unpublished)	13.5	0.0705	77.6	0.008	0.215	12	**0.0092**	1	612	244	0.72	2.21	**3.27×10^{-5}**
CP | Gelatin-0.02 M CsCl [6]	1.7	0.3385	87.5	0.25	0.15	7.7	**0.0006**	2	340.7	96.8	0.0005	0.25	**2.15×10^{-7}**
Cu|PEP2-Na/EMIM:TFSI [7]	17.3	0.0991	11.5	22.5	0.1886	0.43	**0.0059**	1	92.6	201	0.0002	0.023	**8.37×10^{-7}**
Li| EC/DMC-$LiPF_6$ [8]	1.35	2242.6	92	2.42	0.61	30	**2.38**	100	1800	3600	18.26	0.99	**1.85×10^{-3}**
Pt| PAAM/MXene-$FeCN^{4-/3-}$ (unpublished)	1.15	982.5	21.8	3.84	1.1	20	**1.08**	10	300	7200	16.9	16.5	**1.02×10^{-4}**

4. Numerical simulation on the parasitic heat loss of an i-TE generator

During the charging and discharging of an i-TE generator in practical thermal environments, parasitic heat loss exists at the packaging envelope, which could cause a reduction in the heat to power efficiency. To quantify the parasitic heat loss and its possible influence, here we present a numerical simulation on the heat transfer within a typical i-TE generator adopted in our previous experiments [1, 2]. The cell is 15 mm×15 mm×1.8 mm in dimension, and is packaged by a PDMS mould which is 2.5 mm in lateral thickness, as shown in Fig. S3.

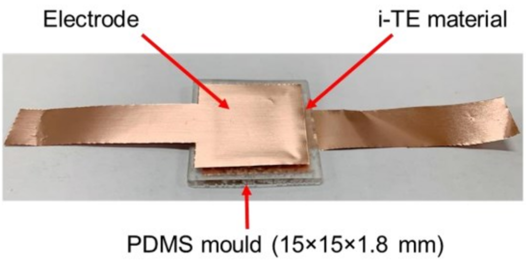

Fig. S3 Picture of the i-TE generator adopted in the numerical simulation

Thermoelectric effects are neglected in the simulation in consideration of the low Peltier effect compared to thermal conduction and low heat to power efficiency. Thermal conductivities of 0.15 and 0.2 W/m·K are specified to the i-TE gel and the PDMS mould, respectively. The top and bottom surfaces of the gel are kept at 44 and 21 °C, respectively, following the thermal conditions in a previous experimental study [2]. Convective boundary conditions are specified at the lateral surfaces of the PDMS mould with a heat transfer coefficient of 5 $W/m^2 \cdot K$. The steady state temperature distribution of the i-TE generator is shown in Fig. S4. Under a temperature difference of 23 °C, the heat flow rate into the top surface is evaluated to be 0.495 W, of which 0.487 W leaves at the bottom surface of the gel and 0.008 W is dissipated at the boundaries of the PDMS mould, resulting in a heat loss ratio of ~2%. It is concluded that the parasitic heat loss generally has a minor effect on the heat to power efficiency of i-TE generators.

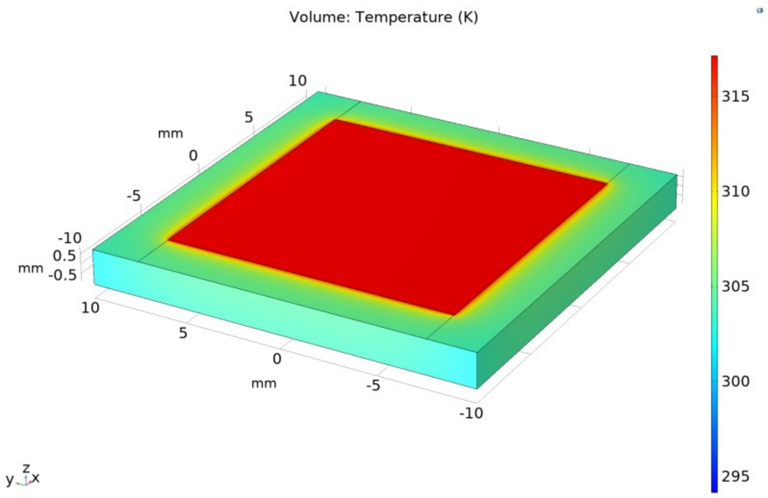

Fig. S4 Steady state temperature distribution of the i-TE generator under specified thermal boundary conditions

REFERENCES

1. Li YC, Li QK, Zhang XB et al. *Adv Energy Mater* 2022; 12: 2103666.
2. Li YC, Li QK, Zhang XB et al. *Energy Environ Sci* 2022; 15: 5379-90.
3. Li YC, Wang SH, Yu M et al. *Adv Energy Mater* 2025; 15: 2402621.
4. Wang SH, Li YC, Yu M et al. *Nat Commun* 2024; 15: 1172.
5. Li YC, Qiu YR, Liao JS, et al. *Nat Commun* 2026; 17: 2209.
6. Xu ZY, Lin SC, Yin YS, et al. *Chem Eng J* 2024; 493: 152734.
7. Yin YS, Lin SC, Xu ZY, et al. *Innov Energy* 2024; 1: 100048.
8. Xu YH, Li ZW, Wu LY, et al. *Nano-Micro Lett* 2024; 16:72.
